# Mucinous Cystadenoma of the Urachal Remnant Successfully Resected by Laparoscopic Surgery: A Case Report

**DOI:** 10.7759/cureus.64790

**Published:** 2024-07-18

**Authors:** Noritoshi Mizuta, Takuya Kikuchi, Hiroshi Ashitani, Nobuya Sano

**Affiliations:** 1 Surgery, Akashi Medical Center, Akashi, JPN; 2 Diagnostic Pathology, Akashi Medical Center, Akashi, JPN

**Keywords:** case report, successfully resected, urachal remnant, mucinous cystadenoma, laparoscopic surgery

## Abstract

A urachal remnant is a disorder resulting from a disturbance in the closure process of the urachus. A 55-year-old man was referred to our hospital for treatment of gallstones. The computed tomography scan revealed a cystic mass in the lower abdomen connecting to the urinary bladder. The preoperative diagnosis was a urachal cyst. Simultaneous laparoscopic cholecystectomy, mass resection, and urachectomy were performed. The mass on the cranial side of the urinary bladder was located on the median umbilical ligament. Both were resected and removed, along with the umbilicus. The postoperative course was uneventful. The histopathological diagnosis was urachal mucinous cystadenoma. There is no sign of a recurrence. A complete resection without damage is especially important for mucinous tumors of the urachal remnant because the injury to the tumor may lead to the development of pseudomyxoma peritonei. Only seven cases of mucinous cystadenoma of the urachal remnant were reported in English literature, and only one of these was treated with laparoscopic surgery. In our case, complete resection was possible by taking advantage of the magnifying effect of laparoscopic surgery. Furthermore, we are able to provide very clear intraoperative images and specimen photographs, which we believe will be useful for readers. Laparoscopic surgery will be beneficial when treating similar cases in the future. However, it should be kept in mind that a safe resection requires careful and meticulous technique.

## Introduction

A urachal remnant is a disorder resulting from a disturbance in the closure process of the urachus, and it may become the primary site of lesions such as cysts, fistulas, tumors, and diverticulum [1]. The pathological classification of urachal neoplasm is based on the 2016 World Health Organization (WHO) classification [2]. Urachal neoplasms are rare because they account for less than 0.5% of urinary bladder neoplasms [3]. Among them, mucinous cystic neoplasms are even rarer [1,4], ranging from benign mucinous cystadenoma to borderline mucinous cystic tumors of low malignant potential to malignant mucinous cystadenocarcinoma [4]. Regarding mucinous cystadenoma, only seven cases have been reported in the English literature [5-8]. Furthermore, laparoscopic surgery was performed in only one case [8]. On the other hand, laparoscopic surgery for the urachal remnant is becoming more common [9]. We encountered a rare case of mucinous cystadenoma of the urachal remnant successfully resected laparoscopically. Laparoscopic surgery has the advantage of being minimally invasive and providing a magnifying effect [9]. However, there is a possibility of dissemination if mucinous tumors are damaged [5,8]. Therefore, it is recommended to exercise caution and employ meticulous techniques.

Urachal mucinous tumors can potentially develop into pseudomyxoma peritonei [5]. Pseudomyxoma peritonei is often associated with the appendiceal and ovarian neoplasms; the other sites, including urachal remnants, are rare [4]. However, precisely because it is a rare condition, it was crucial that we were able to remove the tumor before symptoms were present.

## Case presentation

**Figure 1:**
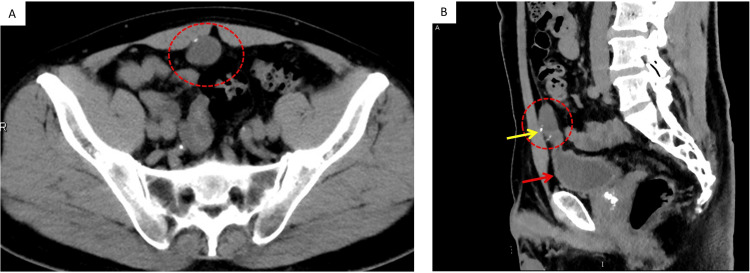
Computed tomography findings. (A) CT (axial plane): a low-density mass in the lower median abdomen behind the rectus abdominal muscle was observed (red dotted area); (B) CT (coronal plane): the mass (red dotted area) was located just cranial to the urinary bladder (red arrow) and seemed to connect to its top. Minimal calcification was observed (yellow arrow).

A 55-year-old man was referred to our hospital for the treatment of gallstones. He sometimes felt epigastric pain. Laboratory data were within the normal range. The tumor markers carcinoembryonic antigen (CEA) and carbohydrate antigen 19-9 (CA 19-9) were also within the normal range. Esophagogastroduodenoscopy (EGD) revealed only chronic gastritis. No ulcer or tumor was found on EGD. Therefore, the epigastric pain was diagnosed as being due to gallstones. However, preoperative computed tomography (CT) not only detected the gallstone but also identified a cystic mass with minimal calcification in the lower abdomen connected to the top of the urinary bladder (Figure 1).

**Figure 2:**
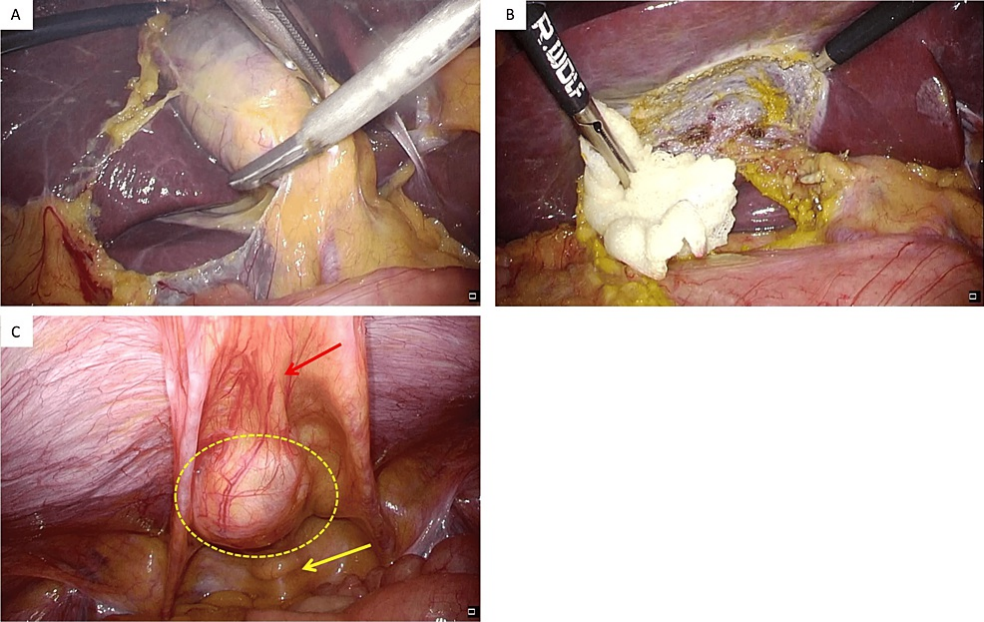
Intraoperative findings. (A) The gallbladder was slightly enlarged; (B) a cholecystectomy was performed; (C) the mass of the lower abdomen (yellow dotted area) was located just cranial to the urinary bladder (yellow arrow) and on the median umbilical ligament (red arrow).

The patient felt no pain in the lower abdomen, and the mass was not palpated. The preoperative differential diagnosis was a urachal cyst. Simultaneous laparoscopic cholecystectomy and mass resection were planned. During preoperative planning, the umbilicus was intended for removal, along with the urachal remnant; therefore, the initial placement of the camera port was on the cranial side of the umbilicus. Next, pneumoperitoneum was started. A 12-mm trocar was inserted at the epigastric region, and a 5-mm trocar was inserted at the right hypochondrium and right flank regions. Initially, a cholecystectomy was performed. Secondly, a mass resection was performed. It was located just cranial to the urinary bladder and on the median umbilical fold (Figure 2).

**Figure 3:**
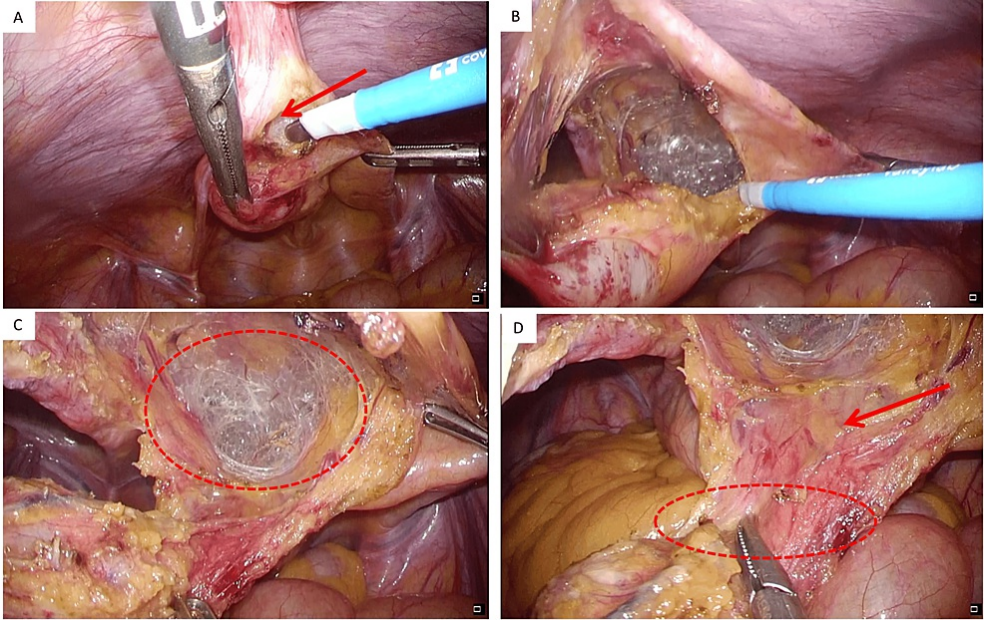
Intraoperative findings. (A) The median umbilical fold and the mass were separated from the posterior sheath of the rectus abdominis muscle (red arrow); (B) the separation continued caudally; (C) the separation was performed up to the urinary bladder. The prevesical space was observed (red dotted area); (D) the urinary bladder wall was recognized (red arrow). The border between the caudal edge of the mass and the top of the urinary bladder was soft (red dotted area).

The median umbilical fold and the mass were separated from the posterior sheath of the rectus abdominis muscle up to the urinary bladder. The border between the caudal edge of the mass and the top of the urinary bladder was soft (Figure 3).

**Figure 4:**
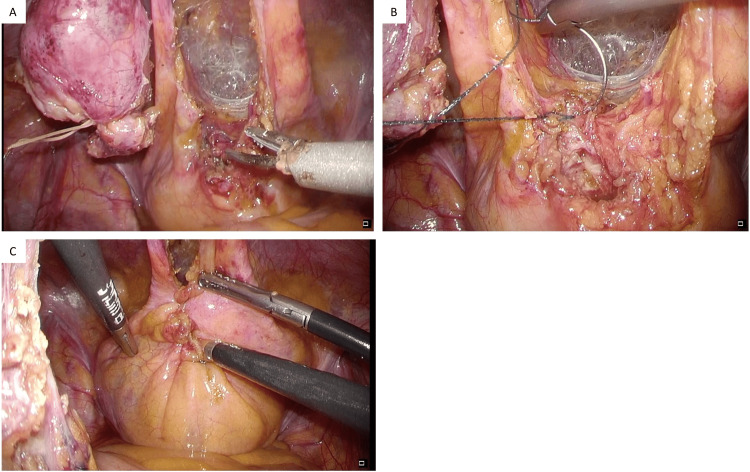
Intraoperative findings. (A) The caudal edge of the mass was ligated with thread, and only the serosa and muscular layers of the urinary bladder connected to the mass were resected; (B) the defective urinary bladder wall was sutured with an absorbable thread; (C) no fluid was observed from the edge of the urinary bladder after the mass resection.

The urinary bladder was filled with 200 mL saline via the Foley catheter; however, the caudal edge of the mass was not enlarged. Therefore, we determined no apparent communication between the mass and the urinary bladder, so partial cystectomy was not performed. The caudal edge of the mass was ligated with thread to avoid spilling the contents, and only the serosa and muscular layer of the urinary bladder connected to the mass was resected. The defective urinary bladder wall was sutured with an absorbable thread. Following the urinary bladder wall repair, a leak test was performed by introducing an additional 100 mL of normal saline solution through the Foley catheter, but no fluid was observed from the edge of the urinary bladder (Figure 4).

**Figure 5:**
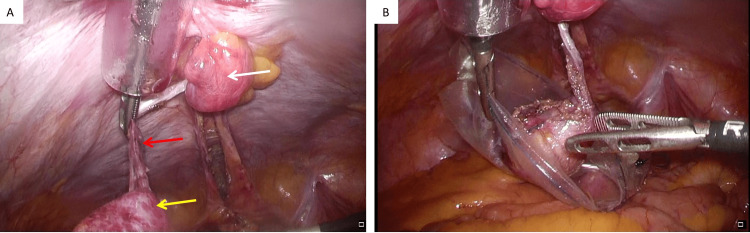
Intraoperative findings. (A) The mass (yellow arrow) and the median umbilical fold (red arrow) were separated from the posterior sheath of the rectus abdominis muscle up to just below the umbilicus (white arrow); (B) the mass was inserted into the bag to prevent spilling out the contents.

Next, the mass and median umbilical fold were separated from the posterior sheath of the rectus abdominis muscle up to just below the umbilicus. The mass was inserted into the bag to prevent spilling out the contents. Finally, the umbilicus was drilled out, and the mass and median umbilical fold were removed in one piece (Figure 5).

**Figure 6:**
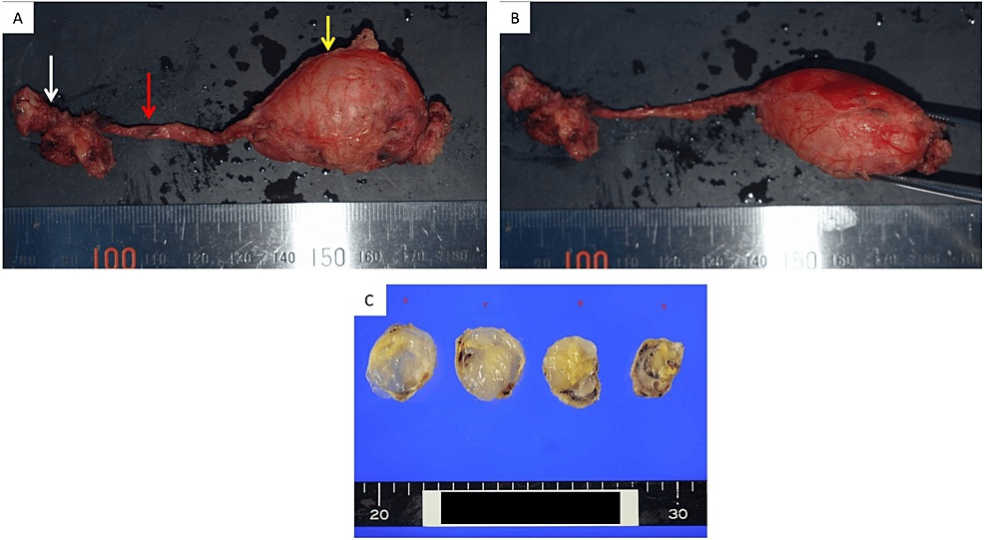
Resected specimen. (A) The umbilicus (white arrow), the median umbilical fold (red arrow), and the tumor (yellow arrow) were completely resected in one piece; (B) the tumor was cystic and had a smooth surface; (C) the content of the tumor was mucous.

The peritoneal defect was not completely closed due to lack of adequate tissue. The operation time was 216 minutes, and the amount of bleeding was 10 mL. The tumor was a cystic mass with a smooth surface, measuring 5.6 x 3.7 cm. When examined from the cut surface, the tissue was divided into a wall structure and a lumen, and the lumen was filled with mucinous components (Figure 6).

**Figure 7:**
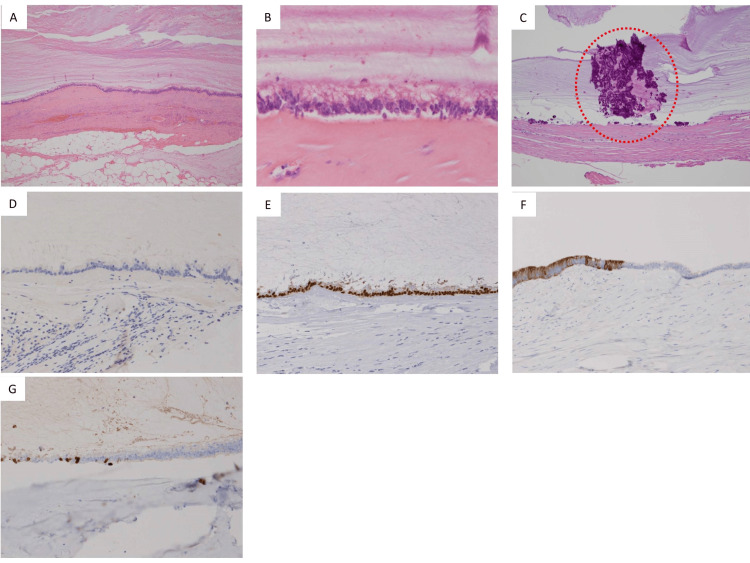
Histopathological findings. (A) H&E staining (low magnification): the wall of the tumor was partially covered with a simple or stratified epithelium; (B) H&E staining (high magnification): mucus production was observed in some of the tall columnar epithelium; (C) H&E staining (high magnification): microcalcification was observed (red dotted area); (D) uroplakin II was negative; (E) CDX2 was positive; (F) CK20 was positive; (G) Ki67-LI was 10%. H&E: hematoxylin and eosin.

A histopathological examination was performed, which revealed a cystic lesion partially covered with a simple or stratified columnar epithelium, and mucus production was observed in some of the tall columnar epithelium. Scattered microcalcification was found within the mucus along the cystic wall. The immunohistochemical stains were performed, and the tumor epithelial cells showed positive results for CDX2, CK7, and CK20 and negative for uroplakin II. The Ki-67 LI was 10%, and the p53 LI was 0% (Figure 7).

The final diagnosis was mucinous cystadenoma arising on the urachal remnant. The gallbladder was diagnosed as chronic cholecystitis. The postoperative course was uneventful, and the patient was discharged nine days after the operation. There are currently no signs of recurrence two years and seven months after the operation.

## Discussion

A urachal remnant may occur in approximately 32% of adults, and it can present as a patent urachus, umbilical-urachal sinus, vesicourachal diverticulum, and urachal cyst [4]. Urachal neoplasms are rare because they account for less than 0.5% of urinary bladder neoplasms [4]. The wall of the urachus consists of three layers of structure, followed by transition epithelium, connective tissue, and residual smooth muscle cells [1]. Urachal neoplasms mostly originate from the epithelium [1]. The epithelial neoplasms arising from the urachus are divided into glandular, non-glandular, or mixed neoplasms [1,2]. Glandular neoplasms are further classified as adenoma, cystic mucinous neoplasms, and non-cystic adenocarcinoma [2]. Cystic mucinous neoplasms include mucinous cystadenoma, mucinous cystic tumor of low malignant potential, mucinous cystic tumor of low malignant potential with intraepithelial carcinoma, and microscopically or frankly invasive mucinous cystadenocarcinoma, based on the 2016 WHO classification [2].

However, the definitive preoperative diagnosis of the urachal neoplasms is difficult [1]. No specific tumor markers exist, considering the low incidence of urachal neoplasms [1]. CEA and CA 19-9 may be elevated in urinary neoplasms, but they are nonspecific and can be elevated in cancers of other origin [1]. In our case, CEA and CA 19-9 were within the normal range. In an imaging study at CT, calcification of the tumor is commonly observed in borderline mucinous cystadenoma but also in mucinous cystadenocarcinoma [1]. The minimal calcification of the tumor at CT was also observed in our case. Another report described the magnetic resonance imaging (MRI) findings of urachal carcinoma as a mass with increased signal intensity on T2-weighted images due to the presence of mucin [10]. However, an MRI was not performed in our case, and it is something that should be seriously considered.

Mucinous cystadenomas are extremely rare. Only seven cases have been reported in the English literature [5-8], and only one has been resected laparoscopically [8]. We have summarized eight cases, including ours, in Table 1.

**Table 1:** Clinicopathological characteristics of mucinous cystadenoma of the urachal remnant. NA: not available; MCA: mucinous cystadenoma.

No.	Author	Year	Age	Sex	Clinical Symptoms	Size (cm)	CT	Place	Pathological Findings	Diagnosis	Surgery	Follow-up Period (Months)
1	Saha et al. [5]	2011	60	Female	Increased frequency of urine	3x3	NA	The space of Retzius anterior to the dome of the bladder	Glandular epithelium that lacked pseudostratified and villous area; no foci of dysplasia	MCA	Excision of mass	48
2	Amin et al. [6]	2014	59	Male	Abdominal mass and hematuria	7	NA	NA	A single layer of mucinous columnar epithelium with no atypia	MCA	Partial cystectomy	97
3	Amin et al. [6]	2014	33	Male	Cyst rupture and abdominal pain	13	NA	NA	A single layer of mucinous columnar epithelium with no atypia	MCA	Excision of mass	11
4	Amin et al. [6]	2014	24	Female	Microhematuria	1.5	NA	NA	A single layer of mucinous columnar epithelium with no atypia	MCA	Partial cystectomy, urachectomy	1
5	Amin et al. [6]	2014	42	Female	NA	5	NA	NA	A single layer of mucinous columnar epithelium with no atypia	MCA	Excision of mass	NA
6	Wang et al. [7]	2016	56	Male	Incidental finding	7.5x5.3x5.2	NA	NA	A single layer of flat epithelium or tall columnar cells with abundant mucin and focal pseudo-stratification; no nuclear atypia	MCA	Excision of mass	12
7	Agnihotri et al. [8]	2020	27	Female	Incidental finding	2.5x2.5x2	NA	The suprapubic region in line with the median umbilical ligament	Pseudostratified mucin-secreting columnar epithelium with no significant nuclear atypia	MCA	Laparoscopic excisional biopsy	NA
8	Our case	2024	55	Male	Incidental finding	5.6x3.7	Well-defined mass with a slight calcification	The caudal side of the bladder in line with the median umbilical ligament	A single layer or several layers of epithelium and mucin-producing high-columnar epithelium. Scattered calcification was found within the mucus along the wall	MCA	Laparoscopic excision of mass and urachectomy combined with cholecystectomy	31

The average age of the patients is 44.5 years old, and the male-to-female ratio is 1:1. The clinical symptoms were frequent urination, hematuria, abdominal pain, and incidental findings. The average size of the tumor was 5.3 cm. Surgical resection is a definitive treatment for mucinous cystadenoma of the urachal remnant, and complete surgical resection leads to a good prognosis [4]. Regarding the surgical method, partial cystectomy was performed in two cases, and urachectomy was also performed in two cases, including our case. Laparoscopic resection was performed in two cases, including our case. However, our case is the only one in which the resected specimen was clearly presented. Furthermore, in our case, the mass was resected en-bloc with the urachus and the umbilicus. The other laparoscopic surgery case was tumor resection only.

The primary treatment strategy for tumors deemed to be urachal is partial cystectomy, urachectomy, and umbilical resection [6]. In general, despite the rarity of urachal cancer originating from the urachal epithelium of the urinary bladder, complete resection of the urachal epithelial tissue is considered necessary for preventing late malignant transformation [9]. It is difficult to determine the exact range of lesions in preoperative imaging studies, such as CT and MRI [9]. Therefore, umbilical resection and partial cystectomy should be considered [9]. Furthermore, in cases of patients older than 30 years of age who have a urachal remnant with hematuria and bladder irritation symptoms, preoperative cystoscopy should be performed to judge the need for partial cystectomy [9]. The other report emphasized the necessity of a preoperative cystoscopy to evaluate whether the carcinoma has reached the urothelium of the urinary bladder [10].

Urachectomy and umbilical resection were also performed in our case, but partial cystectomy was not performed. We planned to perform a partial cystectomy preoperatively. However, the border between the tumor and the bladder was soft, and the caudal edge of the urinary bladder was not enlarged even after the normal saline injection. Therefore, we determined no apparent communication between the mass and the urinary bladder, so partial cystectomy was not performed. We decided to resect only the serosa and the muscle layer of the urinary bladder wall connected to the tumor. However, since a definitive diagnosis had not been made during the operation, it may have been necessary to perform a resection until the bladder lumen could be confirmed. Furthermore, if a preoperative cystoscopy had been performed and no abnormal findings had been found, there would have been no need to hesitate in making this decision. This is also something that should be reflected upon in our case. If preoperative cystoscopy reveals a tumor in the urachal tract, cystectomy is indicated, but whether partial or radical resection should be performed is controversial [11].

The reports of the laparoscopic procedure for the urachal remnant increased [9,12], and the single-incision technique was also reported [12]. The advantages of laparoscopic surgery are magnification and minimal invasiveness. Maemoto et al. summarized 210 operative cases for urachal remnants in Japan and reported that 54.7% (115 of 210) of the cases underwent laparoscopic surgery [9]. However, many of the laparoscopic cases were urachal sinus; on the other hand, the open procedure was often performed for urachal cysts, patent urachus, and vesicourachal diverticulum [9]. Depending on the type of urachal remnant, surgical methods may also need to be considered [9]. In any case, the utmost priority is a complete resection of the lesion without damage. If the lesion is a mucinous tumor, as in our case, injury to the lesion can cause mucus cells to spill into the peritoneal cavity, leading to the development of pseudomyxoma peritonei [8,9]. Therefore, delicate and careful techniques are especially required for the laparoscopic procedure. In our case, the surgery was performed with attention to the following points: cautious use of forceps, ligation of the caudal side of the tumor to prevent leakage of contents, and placing the tumor in a bag. As a result, laparoscopic resection was successfully performed. However, if it is determined that there is a risk of damage to the lesion, it is necessary to decide to proceed to open surgery without hesitation.

Regarding pathological examination, mucinous cystadenoma in the urachal remnant was a well-circumscribed cystic filled with abundant mucin [4]. Histologically, the epithelial lining on the cyst wall was a single layer of mucinous, cuboidal, or columnar cells with no atypia [4,6]. Focal pseudostratification was occasionally observed [4,7,8], and mitosis was rare [4]. Chronic lymphocytic infiltration, multinucleated giant cell reaction, fibrosis, and cyst wall calcification are frequently identifiable [4]. On immunohistochemical staining, most urachal mucinous cystic tumors are positive for CK20 (100%), CDX2 (80%), and CK7 (30%) and negative for β catenin, estrogen receptor, and progesterone receptor [4,6]. The pathological findings in our case were similar to those described above.

Furthermore, as mucinous cystadenoma progresses to mucinous cystadenocarcinoma, which has a very poor prognosis, we think it was of great significance that we were able to remove the tumor successfully at this early stage.

## Conclusions

Mucinous cystadenoma of the urachal remnant is a very rare disease. However, there is potential for it to develop into pseudomyxoma peritonei. Therefore, it is crucial that we were able to remove the tumor before symptoms were present.

The golden standard of treatment is surgical resection. If careful technique is possible, laparoscopic surgery may be an option because of its advantages of magnified vision and minimal invasiveness. However, due to the limited number of reports, it is crucial to gather additional cases in the future, including conducting long-term follow-ups.
